# RISK AND PREVENTIVE FACTORS FOR TRAFFIC ACCIDENTS: ANALYSIS OF CHILDREN’S PERCEPTION USING THE EDUTHERAPEUTIC METHOD

**DOI:** 10.1590/1984-0462/2020/38/2018281

**Published:** 2020-07-13

**Authors:** Carla Kalline Alves Cartaxo Freitas, Manuel Alves Rodrigues, Viviane Santos Fontes, Maria do Socorro Claudino Barreiro, Ana Carla Ferreira Silva dos Santos, Shirley Verônica Melo Almeida Lima, Natália de Jesus Leal, Kássia Patrícia Alves Cartaxo, Edilene Curvelo Hora Mota

**Affiliations:** a Universidade Federal de Sergipe, Lagarto, SE, Brazil.; b Escola Superior de Enfermagem de Coimbra, Coimbra, Portugal.; c Faculdade Santa Maria, Cajazeiras, PB, Brazil.

**Keywords:** Child, Accidents, traffic, Risk factors, Accident prevention, Perception, Adolescent, Criança, Acidentes de trânsito, Fatores de risco, Prevenção de acidentes, Percepção, Adolescente

## Abstract

**Objective:**

To describe children’s perception of risk and preventive factors related to traffic accidents using the Edutherapeutic Method.

**Methods:**

This is a qualitative descriptive study carried out with 173 students from public schools aged seven to 14 years in Lagarto, Sergipe, Brazil. Data were collected in the second half of 2014. The first stage consisted of an activity with drawing/writing sheets in all classes selected by the Nursing undergraduate students. Next, the children answered a questionnaire on sociodemographic data. The qualitative analysis of the expressive language of the children’s drawings generated two categories: positive and negative factors for the prevention of traffic accidents and their subcategories.

**Results:**

The children’s perception regarding preventive and risk factors for traffic accidents was considered adequate according to other studies found in the literature on the same subject. The drawings and descriptions were used later to provide the students with a better understanding of these factors.

**Conclusions:**

The study lends support to educational activities and interventions about prevention with schoolchildren. This is one of the main goals proposed by Brazil in the National Plan of Action for Road Traffic Safety for the decade 2011-2020.

## RESUMO

**Objetivo:**

Descrever a percepção de crianças em relação aos fatores de risco e de prevenção relacionados aos acidentes de trânsito pelo Método Eduterapêutico.

**Métodos:**

Estudo descritivo, com abordagem qualitativa, realizado com 173 estudantes da rede pública do município de Lagarto, Sergipe, com idades entre sete e 14 anos. A coleta de dados ocorreu no segundo semestre de 2014 e se iniciou com a aplicação da ficha do desenho/escrita em todas as turmas selecionadas, pelas alunas do curso de Enfermagem, seguida da aplicação de um questionário sobre dados sociodemográficos. Após análise qualitativa da linguagem expressiva dos desenhos das crianças, emergiram duas categorias: fatores positivos e negativos para a prevenção de acidentes e suas subcategorias.

**Resultados:**

As crianças possuem percepção considerada adequada em relação aos fatores preventivos e de risco para acidentes no trânsito, de acordo com a descrição na literatura a respeito da temática. Os desenhos e as descrições foram utilizados posteriormente para trabalhar esses fatores com os próprios alunos.

**Conclusões:**

O estudo fornece subsídios para intervenções e atividades educativas de prevenção voltadas a escolares, o que se constitui uma das metas importantes da proposta do Brasil no Plano Nacional de Redução de Acidentes e Segurança Viária para a década 2011-2020.

## INTRODUCTION

Traffic accidents are responsible for a high number of victims and represent a major public health problem. However, this topic is neglected by governments and the general population.^1^ According to the Global Status Report on Road Safety, traffic accidents cause more than 1.3 million deaths and 20-50 million injuries each year, resulting in a huge impact on the health and development of the country.^2^

Faced with this problem, the United Nations General Assembly has declared the 2011-2020 period as the Decade of Action for Road Safety, aimed at reducing the number of road deaths by 50% and saving about five million lives. ^3^ After five years, 88 countries have reduced the number of road fatalities. Nevertheless, there is still deep and persistent concern about countries that have increased the number of fatalities over the same period.

The worldwide number of road deaths remains high (1.25 million deaths per year), with the greatest incidence in low-income countries, such as Brazil, which, according to the National Mortality Information System (SIM), had a total of 38,265 deaths resulting from traffic accidents in 2016.^4^ ^,^ ^5^ In 2014, 4,665 accidental deaths of children and adolescents aged zero to 14 years were reported in Brazil - traffic accidents were the main cause, accounting for 38% of fatalities.^6^ Thus, preventing traffic accidents is an ongoing challenge and should, therefore, be taken seriously.^7^

Childhood must be understood as an autonomous social category, as it has its own dynamics within society. Time and space are fundamental for discoveries and a healthy life. However, when associating the physical, perceptual, cognitive, social, and attitudinal conditions of children in the current urban space, we realize how vulnerable they are to the risks of traffic accidents.^8^

The statistical data on traffic safety for children are worrying, and it is necessary to adopt educational strategies for accident prevention. Traffic education is a pedagogical process that aims to shape and transform the attitudes of human beings toward a collective and healthy life, seeking to improve the quality of life through responsible behaviors in order to reduce these events.^8^

Drawings and graphic symbols are some of the first forms of spontaneous communication used by children. Thus, this study adopted the Edutherapeutic Method, a technical-educational, psychopedagogical, and therapeutic tool developed by Rodrigues, in Portugal^9^, which is suitable for children with specific educational and health needs. This line of research has been conducted in Portugal and has allowed the assessment of perceived health and its interventions.^9^ ^,^ ^10^ By using drawings as a research tool, the present study aimed to describe children’s perception of risk and preventive factors related to traffic accidents using the Edutherapeutic Method. Children are expected to have reasonable knowledge about risk and preventive factors for traffic accidents, especially regarding the use of safety devices, traffic lights, and crosswalks.

## METHOD

This is a qualitative descriptive study that analyzed the expressive language of children’s drawings. This analysis is known as the Edutherapeutic Method, which investigates the informative and recreational power through drawing/writing/image, joining children’s cognitive and emotional blocks, as well as facilitating interaction between children, educators, and health professionals.^10^

The data collection instrument was an adapted drawing/writing sheet^9^ distributed to a total of 173 children attending the 3^rd^, 4^th^, and 5^th^ grades of elementary school from two public schools in the city of Lagarto, Sergipe, Brazil, in 2014. Lagarto is the most populous municipality in the inland of Sergipe and the third largest in the state, with an estimated population of 103,576 inhabitants^11^ and a total of 12,430 cars, that is, one car for every eight inhabitants.^12^

With the increasing number of vehicles traveling on urban roads, the city and its population face mobility challenges and problems found in other Brazilian cities, including limited public transport (which was implemented in the city of Lagarto in 2016), excess vehicles, and the resulting congestion of roads and disrespect for pedestrians.

The schools were selected by simple randomization in the site *random.org*. At the end of the process, six classrooms were chosen, with a total of 173 students. This total was defined by convenience, as the sample is non-probabilistic. The study population consists of 89 boys and 84 girls, aged 7 to 14 years (with two 7-year-old children attending the 3^rd^ grade). Despite the age group evaluated, we did not differentiate between the information collected from children and adolescents because the criterion chosen by this study was the random selection of classrooms and not age.

We emphasize that children participate in school activities related to traffic education every year, during the week dedicated to this theme, since article 76 of the New Brazilian Traffic Code states that traffic education should be promoted in preschool, elementary school, and high school. The month selected for the national road safety week is September, when most Brazilian schools address the issue in the classroom. The questionnaire was administered one month after this event.

The inclusion criteria to participate in the research were: knowing how to read and write; completely and legibly complete the drawing/writing sheet. There were no exclusion criteria.

The drawing/writing sheet was distributed, and the questionnaire composed of closed questions was administered by previously trained undergraduate Nursing students from Universidade Federal de Sergipe (UFS), located in Lagarto, Sergipe. The activity took place in the same classrooms the students attended, and they had 30 minutes to finish.

The drawing/writing sheet was divided into four areas: two at the top, in which the children made drawings of actions that prevent and cause traffic accidents; and two at the bottom, where they wrote the message related to their drawings. We underline that the analysis only considered the written content, and the researcher did not perform a psychodynamic analysis of the drawing. The themes expressed were classified and ranked according to priority areas for reducing the risk of traffic accidents, according to the Brazilian Traffic Code (*Código de Trânsito Brasileiro* - CTB),^13^ based on the analysis of the drawings and the evaluation of four experts in the field, selected for their experience in health education for children and traffic education.

After transcribing the data into a Word document, we used the Content Analysis proposed by Bardin.^14^ This analysis involves classifying topics relevant to the research question and the student’s oral/written production, allowing us to define *a priori* and *a posteriori* analytical categories. Categorization is a fundamental aspect of content analysis and consists of a back and forth assessment between the theory and the material under analysis, which usually leads to various developments and reworkings of the material until the analytical categories are defined.^14^

In this process, the record units were coded to allow only gender identification; for example, in the code “F38m,” “F” means drawing/writing sheet; the number 38 is the subject’s interview order; and “m” corresponds to male. Although some drawings allow an immediate interpretation, we adopted the strategy indicated by the literature - double-code reading (drawing and writing) -, valuing the meaning the child attributes to their drawing, expressed in writing.^15^

The research was authorized by the Secretariat of Education of Sergipe, the administration of the schools, and the Research Ethics Committee (REC) of UFS, since it involves human beings, under opinion No. 298,534, respecting all ethical-legal requirements, as per Resolution No. 466/12, especially those regarding the consent of the children’s parents and the involvement of teachers.

## RESULTS

All 173 students completed the test. Among them, most were ten-year-olds (53-30.6%), followed by nine-year-olds (43-24.9%), and 11-year-olds (42-24.2%). The ages with the least participants were 12 years (3-1.7%), seven years (2-1.2%), and 14 years (1-0.6%). Table 1 describes the school travel mode reported by the students. Most children used some type of transport, followed by walking.

After analyzing the expressive language of the children’s drawings, the following thematic categories and subcategories emerged:

- Positive factors for the prevention of traffic accidents according to children’s perception: preventive driver behavior, preventive pedestrian behavior, preventive behavior of all road users, road signs, engineering, and proper traffic enforcement.
- Risk factors for accidents according to children’s perception: risky driving behavior, motorcyclist risk behavior, pedestrian and cyclist risk behavior, lack of road signs, and inadequate traffic enforcement.

**Table 1:** Characterization and comparison of the travel mode used by public school students, according to the experimental group and the control group.

School travel mode	Experimental group n=90 (%)	Control group n=83 (%)
Walking	31 (35.2)	24 (29.3)
Walking accompanied by an adult	18 (20.5)	20 (24.4)
Some type of transport	38 (43.2)	36 (43.9)
Other	1 (1.1)	2 (2.4)

Table 2 presents the preventive factors for traffic accidents perceived by the children based on the analysis of their drawings. In this category, children show the importance of preventive behavior in road traffic, be it by wearing a seat belt or by not consuming alcohol while driving, and mention that these behaviors must be adopted not only by the driver but also by the pedestrian who should cross the street on the crosswalk after looking both ways. Traffic behaviors that belong to all road users include respecting road signs and traffic lights, paying full attention to the surroundings.

**Table 2:** Children’s perception of preventive factors for traffic accidents.

Category: Positive factors for the prevention of traffic accidents, according to children’s perception.		
Subcategory	Drawing	Indicators (phrases)
Preventive driver behavior	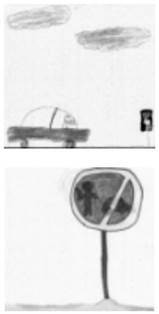	*“Using a seat belt” (F171f).* *“Do not drive when drinking alcohol” (F82m).*
Preventive pedestrian behavior	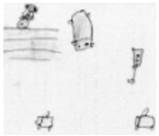	*“She should cross the street on the crosswalk” (F59f).* *“Look both ways before crossing the street” (F31m).*
Preventive behavior of all road users	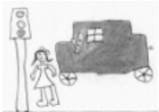	*“Respect traffic lights and always stop when someone is crossing the street” (F35f).* *“Here I made a drawing of him paying attention to traffic” (F36f).*
Road signs, engineering, and proper traffic enforcement	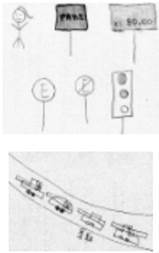	*“People have to look at the signs to avoid accidents” (F40f).* *“Here are the 3 traffic officers. Watching”(F172f).*

Table 3 shows the children’s perception of negative factors for the prevention of traffic accidents based on the analysis of their drawings. The children were very clear when demonstrating risk behaviors, such as high speed, disregard for road signs, drinking while driving, and even risky maneuvers performed by drivers. With respect to risk behavior, pedestrians and cyclists were shown displaying the behavior of many children in traffic, who run between cars and ride bikes holding onto a moving vehicle. However, the children’s perception goes beyond road users, as they also point out the lack of road signs and adequate traffic enforcement.

**Table 3:** Children’s perception of risk factors for traffic accidents.

Category: Risk factors for traffic accidents, according to children’s perception.		
Subcategory	Drawing	Indicators (phrases)
Risky driving behavior	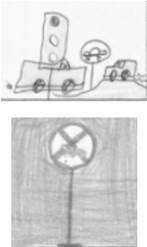	*“The car is at high speed and runs the red light” (F50m).* *“Driving at high speed and drinking” (F64m).*
Motorcyclist risk behavior	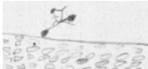	*“A motorcycle doing a wheelie and falling” (F176m).*
Pedestrian and cyclist risk behavior	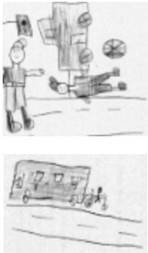	*Do not run in front of the car, playing on the road is dangerous” (F33f).* *“In this picture, the guy is on a bike, and he’s holding onto the bus” (F21m).*
Lack of road signs and inadequate traffic enforcement	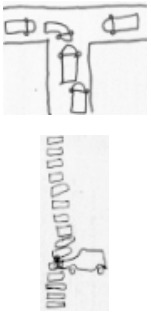	*“(…) I wanted to show that without traffic lights and yellow lines, many accidents can happen and that without separating the cars from those who come without looking, there are many accidents and the bad model of the other drawing” (F70f).* *““There is no road sign there” (F54m).*

## DISCUSSION

The results provide support for educational activities and interventions about prevention aimed at schoolchildren, which is one of the goals proposed by Brazil to the United Nations (UN) in the Life in Traffic Project regarding the National Plan of Action for Road Traffic Safety for the decade 2011-2020. This plan was a worldwide agreement to reduce the number of traffic accident deaths.^3^ In it, the Brazilian initiative addresses surveillance, prevention of road traffic injuries and deaths, and health promotion.^16^

When analyzing the children’s drawing/writing sheets on accident prevention factors, the emphasis was placed on preventive driver behavior, also known as defensive or safe driving, which is considered the best way to drive and behave in traffic. This driving style allows the individual to recognize dangerous situations in advance and predict what may happen to their passengers, their vehicle, and/or other road users.^17^

Among the preventive behaviors, children highlighted speed control to avoid accidents, as well as the importance of using protective equipment, such as seat belts. Studies show that driving at high speed is one of the behaviors that jeopardize traffic safety and that measures, such as seat belt use, have been taken to prevent accidents or reduce possible injuries when they occur.^18^ ^,^ ^19^

A new characteristic related to preventive driver behavior is the prohibition of alcohol consumption while driving. This is one of the main protective factors for the prevention of traffic accidents.^20^ Alcohol consumption while driving has been reported as the cause of 30% of all traffic accidents and approximately 70% of those resulting in severe or fatal injuries.^21^

Regarding pedestrians, the participating children mentioned the importance of crossing the street at the crosswalk and looking both ways before and while crossing, (Table 2). Half of all traffic deaths worldwide occur among the most vulnerable road users: motorcyclists (23%), pedestrians (22%), and cyclists (4%). In 2015, the number of pedestrian deaths in Brazil reached 4.6/100 thousand inhabitants,^2^ and the attention given to the needs of these people, who represent 49% of all road traffic deaths worldwide, has been insufficient.

At present, the answer given to traffic problems is focused on changing the behavior of all road users, so it is necessary to understand the main determinants and risk factors for aggressive traffic: drivers whose behavior is inappropriate and undesirable; inattentive pedestrians or those who disobey road signs; neglectful, poor, careless, and pointless surveillance; inadequate, bumpy, poorly designed, poorly marked, and poorly lit roads and streets; and vehicles deemed unsafe for traffic.^22^ Once the various factors that lead to aggressive traffic are understood, it will be possible to devise strategies for its prevention.^23^

Participants demonstrated the relevance of road signs for accident prevention, as well as the need for traffic enforcement and investment in traffic engineering to make traffic faster, safer, and more efficient.^24^ On the other hand, one of the main problems is the drivers’ lack of attention to road signs, which causes a great number of accidents.^24^

The change in behavior of road users is directly related to enforcement, which must be regular and proficient, but remains inefficient and in need of improvement.^25^

Risk factors are related to human factors (people’s behaviors and actions); road-environmental features (related to the surrounding road or environment); vehicles (their design or mechanical failure); institutional characteristics (laws, method of enforcement, and investment in transportation and safety); and socioeconomic aspects.^26^

The main factors involving the driver are: haste, alcohol consumption, speeding, neglect, recklessness, disregard for road signs, impatience, and inability to manage risk.^27^ Some drawings and phrases produced by the participants addressed the fact that driving under the influence of alcohol was prohibited. This behavior is considered one of the main causes of traffic accidents for reducing the ability to react appropriately to stimuli (reflexes), decreasing peripheral vision, changing body control, increasing aggression, and causing sleepiness and drunkenness. Alcohol affects all tissues and has a significant impact on the nervous system.^28^

The CBT Brazilian Traffic Code amendments, through Law No. 12,760/2012, propose penalties to the individuals driving with any alcohol concentration per liter of blood or alveolar air^29^. However, 60.3% of drivers have not yet changed their behavior after the introduction of this new law.^28^

Concerning motorcyclists, the students described the very dangerous maneuver of balancing the motorcycle on one wheel in their drawings. In Article 175, the CTB considers the use of a vehicle to demonstrate or perform dangerous maneuvering, such as sudden jerking, skidding, or braking with tire sliding or dragging, a serious violation.^30^

As to cyclists, one risk attitude highlighted by the children was holding onto a moving vehicle. Bicycle accidents are not very prominent as a cause of death, but it is important to emphasize that cyclists should be considered more vulnerable than other drivers.^8^ Accidents involving cyclists are related to the use of the bicycle for leisure activities or to cycle to school, lack of bike lanes, poor road conditions, lack of recreational areas, ignorance of laws, lack of balance, underestimation of risks, and lack of protective equipment.^8^

Finally, this study identified that children have an adequate perception of preventive and risk factors for traffic accidents. Thus, this study reached its objective: analyze the expressive language of drawings, using a drawing/writing sheet, and identify the preventive and risk factors for traffic accidents perceived by the child.

The data collection instrument revealed its technical-educational and research potential, facilitating the interaction between educators, health professionals, children, and technical-educational tools, which are useful for planning and implementing actions, even with the limitation regarding the follow-up period of the study, given that the students were not monitored in the subsequent months to ascertain the long-term influence of the educational activity. Therefore, it is important to expand the use of the method in Brazil and incorporate it in public health policies in schools, training professionals involved in health care and child education, to promote appropriate traffic behavior.

The set of thematic axes found in this research contributes to preventive educational practices related to traffic accidents, aimed at children in school environments, representing one of the principal goals proposed by Brazil, according to the National Plan of Action for Road Traffic Safety for the decade 2011-2020. There was no difficulty in implementing the method in the Brazilian context. Adopting this method is essential to promote preventive traffic education for children. We believe that the method can be used in new educational contexts, allowing immersion in the local culture, while creating a space for dialog and learning.
