# ERRATUM

**DOI:** 10.1590/1984-0462/2020/38/2018281erratum

**Published:** 2021-07-16

In the manuscript “Risk and preventive factors for traffic accidents: Analysis of children’s perception using the edutherapeutic method”, DOI: 10.1590/1984-0462/2020/38/2018281, published in the Rev Paul Pediatr. 2020;38:e2018281, on page 7:

**Where it reads:**

Funding

This study did not receive funding.

**It should read:**

Funding

This study was ﬁnanced in part by the Coordenação de Aperfeiçoamento de Pessoal de Nível Superior - Brasil (CAPES) - Finance Code 001.

